# Hepatoid Carcinoma of the Pancreas: A Case Report and Review of the Literature

**DOI:** 10.1089/crpc.2015.29001.nlw

**Published:** 2015-11-01

**Authors:** Noelle L. Williams, Joshua D. Palmer, Voichita Bar-Ad, Pramila Rani Anné, Ashwin R. Sama, Jonathan C. Weinstein, Miguel L. Rufail, Charles J. Yeo, Mark D. Hurwitz

**Affiliations:** ^1^ Department of Radiation Oncology, Bodine Center for Cancer Treatment, Sidney Kimmel Cancer Center and Sidney Kimmel Medical College, Thomas Jefferson University, Philadelphia, Pennsylvania.; ^2^ Department of Medical Oncology, Thomas Jefferson University Hospital, Philadelphia, Pennsylvania.; ^3^ Department of Radiology, Thomas Jefferson University Hospital, Philadelphia, Pennsylvania.; ^4^ Department of Pathology, Anatomy and Cell Biology, Thomas Jefferson University Hospital, Philadelphia, Pennsylvania.; ^5^ Department of Surgery, The Jefferson Pancreas, Biliary, and Related Cancer Center, Thomas Jefferson University, Philadelphia, Pennsylvania.

**Keywords:** ectopic, hepatocellular, hepatoid, pancreas

## Abstract

**Background:** Hepatoid carcinoma (HC) is a rare extrahepatic malignancy that shares many morphological and serological features with hepatocellular carcinoma. HC has been reported to arise from several organs that are derived from the foregut endoderm, including the stomach, gallbladder, and pancreas. We present a case of an elderly man with hepatoid adenocarcinoma of the pancreatic head with duodenal invasion, presenting with pancreatitis and a gastrointestinal bleed. With only 23 reported cases at the time of our literature search, we discuss the presentation, histopathology, and management of such a rare disease.

**Case presentation:** A 71-year-old man presented initially with abdominal pain and was treated conservatively for pancreatitis. Four months later, he presented with melena and anemia. His examination was noncontributory. Esophagogastroduodenoscopy revealed a friable ampulla of Vater, and a CT scan of the abdomen showed a 4.5 cm pancreatic head mass. Fine needle aspirate revealed an epithelioid neoplasm with hepatoid morphology. Serum α-fetoprotein was normal. Surgical resection confirmed hepatoid adenocarcinoma of the pancreas with positive lymphadenopathy and negative margins. There was no radiographical or gross evidence of distant spread. Observation and adjuvant gemcitabine were discussed as possible options. The patient elected to receive care closer to home and will continue surveillance imaging.

**Conclusion:** With only 23 reported cases, pancreatic HC represents a rare entity within gastrointestinal oncology. There is no clear postoperative adjuvant standard therapy for this likely heterogeneous group of tumors. Although surgical resection is the mainstay of upfront treatment, metastatic disease to the lymph nodes or liver portends a poor prognosis and may warrant treatment such as transarterial embolization, chemotherapy, or radiotherapy.

## Introduction and Background

Hepatoid carcinoma (HC) of the pancreas is a rare extrahepatic neoplasm.^1–3^ Most clinicopathological information has been described through isolated case reports, and thus, the true incidence has not been elucidated. The first case of a pancreatic HC was described by Hruban et al.^4^ The terminology “hepatoid” refers to an ectopic “liver-like” tumor having morphological features similar to those of hepatocellular carcinoma (HCC).^1^ Although this report focuses on pancreatic HC, variations of this tumor can be located in several organs that share their derivation from the foregut endoderm, including the stomach, gallbladder, and pancreas.^2^ Given the location, patients typically present with symptoms of epigastric pain or obstruction.

Microscopically, pancreatic HC is composed of polygonal sheets of cells with abundant cytoplasm and centrally located nuclei. Occasionally, bile canaliculi and actual bile formation are visualized. Tumor cells are organized into glandular, medullary, or trabecular patterns. Many tumors, however, demonstrate mixed architectural patterns.^1^ Histology often reveals “pure form” morphology, which can exist in association with other morphologies such as neuroendocrine carcinomas. Like HCC, HC has historically been associated with elevated serum α-fetoprotein (AFP) levels.^1–3^ Immunohistochemistry (IHC) also reveals similarities to HCC with positive cytokeratin (CK), keratin, albumin, α-1-antitrypsin protease inhibitor, and transferrin among others.^2^

Although no specific diagnostic criteria have been developed for the diagnosis of pancreatic HC, morphology and IHC similar to HCC are required. With no distinguishable primary, the CK panel (CK18, 19, and 20) can be useful at distinguishing primary HCC from pancreatic HC. Normal or neoplastic hepatocytes (in the case of HCC) are typically CK18+, CK19−, and CK 20−. HC differs from CK19 positivity and CK20+/−.^3^

We present the case of an elderly man with primary HC of the pancreatic head with duodenal invasion presenting with pancreatitis and a gastrointestinal bleed. We also discuss the presentation, histopathology, and management of such a rare disease.

## Presentation of Case

A 71-year-old man presented to an outside hospital with abdominal pain and was treated conservatively for pancreatitis. His comorbidities included hypertension and hyperlipidemia. He had no previous abdominal surgeries and denied a history of liver disease, gallstones, or alcohol consumption. He was discharged there after conservative management and presented to our hospital 4 months later with several days of melena and lightheadedness. His hemoglobin was 9 g/dL from a baseline of 14 g/dL and he had melena on digital rectal examination. His vital signs were stable. His abdomen was nontender with no palpable hepatosplenomegaly or masses.

He was initially evaluated with an esophagogastroduodenoscopy (EGD) that revealed a prominent, edematous, and friable ampulla of Vater. There was a small oozing ulcer at the ampulla, but with no active bleeding. Colonoscopy was negative for additional bleeding masses. A CT scan of the abdomen with contrast showed a 4.5 cm enhancing mass along the anterior pancreatic head indenting the second portion of the duodenum.

**FIG. 1.**
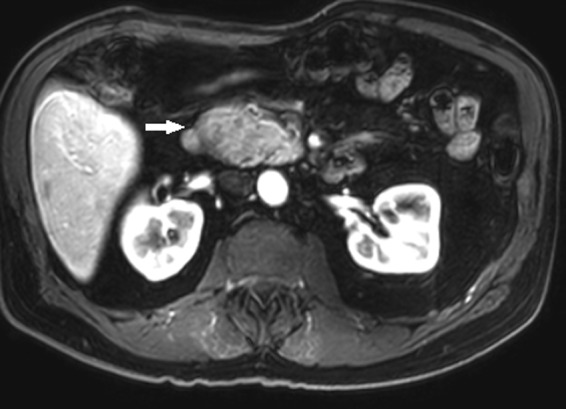
T1-weighted arterial phase contrast-enhanced axial MRI demonstrates heterogeneous enhancement of the well-defined mass (arrow).

**FIG. 2.**
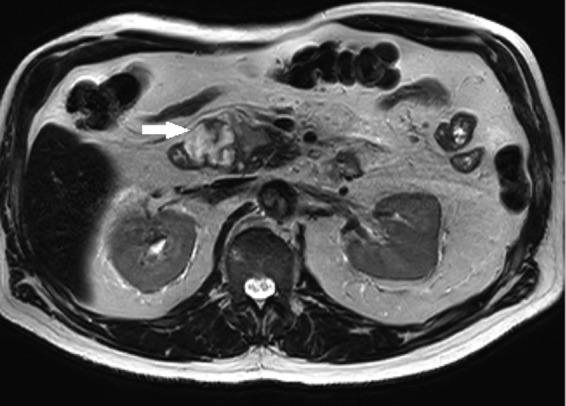
T2-weighted axial MRI demonstrates a well-defined mass (arrow) with high T2 signal components that has borders approximating the second portion of the duodenum and the uncinate process.

Further contrast MRI scan of the abdomen (Fig. 1) revealed sharp margins of the heterogeneously enhancing mass of the pancreatic head and uncinate process abutting the second portion of the duodenum. The lesion demonstrated high signal intensity on T2 sequence (Fig. 2) with hyperenhancement on the postcontrast phase and restricted diffusion. Imaging characteristics were felt to be most consistent with a gastrointestinal stromal tumor.

Pertinent laboratory values include an AFP of 0.6 ng/mL (normal <7.7 ng/mL), cancer antigen 19-9 of 13 U/mL (<35 U/mL), and carcinoembryonic antigen of 3.4 ng/mL (<4.7 ng/mL). Chromogranin A was not measured. Liver function tests were within normal limits. A repeat EGD with endoscopic ultrasound and fine needle aspirate revealed discohesive, large pleomorphic cells in monolayers resembling an epithelioid neoplasm with hepatoid morphology. The cells were positive for CK AE1/AE3, hepatocyte-specific antigen, and C-KIT. The cells stained negative for synaptophysin and chromogranin.

Pylorus-preserving pancreaticoduodenectomy and cholecystectomy were performed. Exploration grossly revealed a well-circumscribed, elliptical shaped mass sitting in the pancreaticoduodenal groove. The mass involved the pancreatic head that bulged into the duodenum. There was no evidence of carcinomatosis, omental implants, or ascites. Grossly, the liver was free from metastatic disease. After meticulous dissection of the pancreatic head and duodenum from the surrounding vasculature, complete resection and successful reconstruction were achieved.

**FIG. 3.**
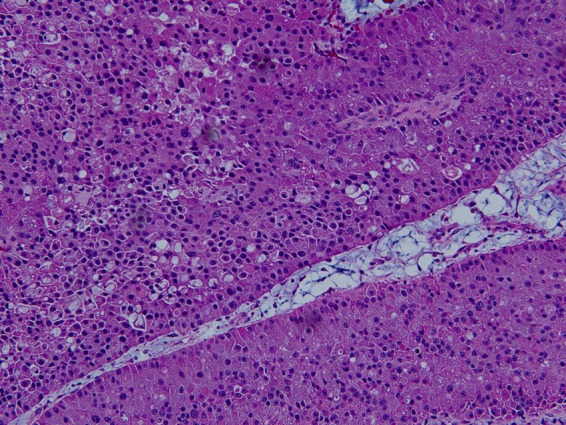
Hematoxylin and eosin low-magnification (20×) microscopic image showing polygonal sheets of cells with abundant eosinophilic cytoplasm and centrally located nuclei.

Histopathological examination of the Whipple resection specimen revealed a moderately differentiated pancreatic hepatoid adenocarcinoma with involvement of the duodenal muscularis propria. The tumor was 5 cm in greatest dimension with lymphovascular and perineural invasion present. Figure 3 depicts the microscopic polygonal sheets of cells with abundant cytoplasm and centrally located nuclei. The excision margins were free of neoplasia and metastatic carcinoma was present in 2 of 17 specimen lymph nodes. Staining for hepatocyte antigen was positive and mucicarmine was focally positive. S100, CEA, AFP, chromogranin, trypsin, arginase, and periodic acid–Schiff (PAS) stains were all negative.

The patient recovered without complication and was evaluated by both medical oncology and radiation oncology. Options presented included observation and gemcitabine, given the adenocarcinoma component. Adjuvant radiotherapy was deferred given the complete local resection with negative margins. The patient elected to receive care closer to home and will continue serial surveillance imaging.

## Discussion and Literature Review

The largest combined analysis of pancreatic HC cases was reported by Kuo et al. and included 23 cases.^1^ The pancreatic body/tail region accounted for 61% of the reported cases and the median tumor size was 6 cm. Of the reported cases, hepatitis B and C were serologically negative in all cases. The median age was 56 years (range 21–80 years) and 70% of patients were male. AFP was elevated in 60% of patients and CA 19-9 and CEA were abnormal in 18% of patients. Pure histology represented 59% of the cases, with the remainder showing mixed histology. The majority of patients (85%) underwent surgical resection upfront and 32% were found to have liver metastases, whereas 21% had lymph node metastases. Overall, 1- and 5-year survival rates were 71% and 40%, respectively (median 13 months).^1^

Embryologically, two theories may explain the origin of this rare tumor. The “ectopic liver” theory assumes that since the liver and the pancreas are both derived from the foregut endoderm, there is a possibility of liver rests in the pancreas. Liver-specific carcinogenesis can then activate ectopic tissue neoplastic transformation. Ectopic liver tissue has also been found in the stomach, gallbladder, and thorax, where HC has similarly been described. A contrast to this theory is the “pancreas-to-liver trans-differentiation” theory. Pancreatic stem cells typically act as suppressors to hepatocyte differentiation genes. Following exposure to carcinogens, these genes can undergo activation, as has been shown in animal models. Proponents of this theory cite the pathologically mixed features of many HCs.^1^

Regarding adjuvant chemotherapy, similar to HCC, there is no standard recommendation for systemic therapy. Adjuvant therapy combinations reported in the literature include gemcitabine, cisplatin combined with irinotecan, and 5-fluorouracil with streptozocin alternating with dacarbazine and adriamycin.^2^ Gemcitabine has shown benefit in pure adenocarcinoma, and these results have been extrapolated to other mixed histologies, including adenosquamous and hepatoid adenocarcinoma. Doxorubicin-based chemotherapy has shown efficacy in unresectable HCC in previous reports, but no improvement in survival.^2^ Petrelli et al. describe a case of metastatic pancreatic HC treated with sorafenib, resulting in more than 7 months of progression-free survival.^3^ Studies investigating adjuvant sorafenib in the treatment of HCC have failed to reveal a survival advantage. Transarterial chemoembolization has also been explored for treatment of liver metastases in several cases.^2^ In the series by Kuo et al., two patients received radiotherapy, but it is unclear at what time interval this therapy was administered and whether these patients were initially unresectable.^1^

Given the rarity of this tumor and the reports described, complete surgical resection remains the treatment of choice for pancreatic HC.^1,2^ Analysis of the reported cases demonstrates the importance of surgical excision. Patients who underwent surgical resection had significantly improved survival. Furthermore, all patients who did not undergo resection died within 1 year of diagnosis.^1^

## Conclusion

This case illustrates an unusual presentation of gastrointestinal bleeding from duodenal invasion of a pancreatic head HC in an elderly male. HC of the pancreas is rare and histopathologically represents a heterogeneous group. Complete surgical resection is paramount and postoperative management is not standardized. Chemotherapy and/or radiation therapy are typically reserved for unresectable, recurrent, or residual disease.

## Abbreviations Used

AFP: α-fetoprotein
CK: cytokeratin
EGD: esophagogastroduodenoscopy
HC: hepatoid carcinoma
HCC: hepatocellular carcinoma
IHC: immunohistochemistry
PAS: periodic acid–Schiff

## References

[1] Kuo PC, Chen SC, Shyr YM, et al. Hepatoid carcinoma of the pancreas. World J Surg Oncol. 2015;13:185 25986692 10.1186/s12957-015-0586-6 PMC4443511

[2] Marchegiani G, Gareer H, Parisi A, et al. Pancreatic hepatoid carcinoma: a review of the literature. Dig Surg. 2013;30:425–433 24281319 10.1159/000355442

[3] Petrelli F, Ghilardi M, Colombo S, et al. A rare case of metastatic pancreatic hepatoid carcinoma treated with sorafenib. J Gastrointest Cancer. 2012;43:97–102 21365478 10.1007/s12029-011-9264-2

[4] Hruban RH, Molina JM, Reddy MN, et al. A neoplasm with pancreatic and hepatocellular differentiation presenting with subcutaneous fat necrosis. Am J Clin Pathol. 1987;88:639–645 3673946 10.1093/ajcp/88.5.639

